# Supplementary material for: Brain MR elastography metrics associated with alterations in learning and memory in people with HIV
Source: Sci Rep. 2026 Sep 19;16:30509. doi: 10.1038/s41598-026-71199-6 (PMC13630558; doi:10.1038/s41598-026-71199-6)
Supplement: Supplementary file 1 [file 41598_2026_71199_MOESM1_ESM.docx]

**Brain MR Elastography Metrics Associated with Alterations in Learning and Memory in People with HIV**

**Supplementary Information**

**Supplementary Tables**

### ***Table 1: Cognitive Domain Assessment***

This table shows the corrected p-values, effect sizes (Cohen's d), and interpretation for various cognitive domains. A p-value less than 0.01 was considered significant.

| **Cognitive Domain** | **HIV+ Mean (Std)** | **HC Mean (Std)** | **Corrected P-Value (q)** | **Cohen's d** |  |
| --- | --- | --- | --- | --- | --- |
| **Learning** | 0.1090 (0.927) | 1.3178(1.102) | **0.0002** | -1.18 |  |
| **Memory** | 0.0611 (0.945) | 1.0287(1.171) | **0.006** | -0.9 |  |
| **Language** | 0.3690(0.825) | 1.102(0.974) | **0.026** | -0.81 |  |
| **Total Score** | -0.38901(3.901) | 3.4733(4.554) | **0.001** | -0.91 |  |
| **Motor Skills** | -0.5759(1.075) | -0.0533(0.907) | 0.369 | -0.53 |  |
| **Speed of Processing** | -0.2490(0.866) | 0.0986(0.856) | 0.966 | -0.4 |  |
| **Attention** | -0.3074(0.595) | -0.2022(0.578) | 1 | -0.18 |  |
| **Executive Function** | 0.2037(0.925) | 0.1816(1.048) | 1 | 0.02 |  |

The following tables detail the interaction p-values from various analyses, including Magnetic Resonance Elastography (MRE), advanced diffusion imaging (NODDI and DTI), and biomarker data.

### **MRE vs Cognitive Scores: Group Interaction Results**

#### **Table 2**: **Correlation (r) per group and Interaction p-value (FDR Corrected) for Viscosity (ϕ) ~ Cognition * Group**

#### **Table 3:**  **Correlation (r) per group and Interaction p-value (FDR Corrected) for G: Stiffness (G) ~ Cognition * Group**

#### **Table 4: Correlation (r) per group and Interaction p-value (FDR Corrected) for Strain ~ Cognition * Group**

### **Diffusion Based Features (NODDI and DTI) vs Cognitive Scores: Group Interaction Results**

#### **Table 5:**  **Correlation (r) per group and Interaction p-value (FDR Corrected) for fISO ~ Cognition * Group**

#### **Table 6:**  **Correlation (r) per group and Interaction p-value (FDR Corrected) for NDI ~ Cognition * Group**

#### **Table 7:** **Correlation (r) per group and Interaction p-value (FDR Corrected) for ODI ~ Cognition * Group**

#### **Table 8:**  **Correlation (r) per group and Interaction p-value (FDR Corrected) for FA (DTI) ~ Cognition * Group**

#### **Table 9:** **Correlation (r) per group and Interaction p-value (FDR Corrected) for MD (DTI) ~ Cognition * Group**

### **Peripheral Marker of Brain Injury vs Cognitive Scores: Group Interaction Results**

#### **Table 10:** Group Interaction P-Values **(FDR Corrected)** and correlation coefficients for **NFL ~ Cognition * Group**

| Cognitive Domain | *r* (HIV+) | *r* (HC) | Interaction *p*-value |
| --- | --- | --- | --- |
| EXEZS | -0.418 | -0.357 | 0.241 |
| SPEZS | -0.443 | -0.354 | 0.181 |
| ATTZS | -0.419 | -0.211 | 0.200 |
| LEAZS | 0.211 | -0.374 | *0.081* |
| MEMZS | 0.223 | -0.411 | *0.061* |
| MOTZS | -0.409 | -0.275 | 0.406 |
| LANZS | -0.364 | -0.124 | 0.190 |
| TOTALZS | -0.352 | -0.418 | 0.435 |

*Note.* *r* represents the Pearson correlation coefficient. Italicized *p*-values indicate a trending interaction (0.05 < *p* < 0.10). HC = Healthy Control.

#### **Table 11:** Group Interaction p-values **(FDR Corrected)** and correlation coefficients for **GFAP ~ Cognition * Group**

| Cognitive Domain | *r* (HIV+) | *r* (HC) | Interaction *p*-value |
| --- | --- | --- | --- |
| EXEZS | -0.359 | -0.267 | 0.388 |
| SPEZS | -0.106 | -0.143 | 0.915 |
| ATTZS | -0.271 | -0.124 | 0.497 |
| LEAZS | -0.198 | -0.283 | 0.996 |
| MEMZS | -0.063 | -0.365 | 0.488 |
| MOTZS | 0.157 | -0.058 | 0.473 |
| LANZS | -0.243 | 0.020 | 0.314 |
| TOTALZS | -0.218 | -0.250 | 0.751 |

*Note.* *r* represents the Pearson correlation coefficient. GFAP = Glial Fibrillary Acidic Protein. HC = Healthy Control. All interaction *p*-values in this set were non-significant (*p* > 0.10).

### **Blood and Vascular Biomarkers vs Cognitive Scores: Group Interaction Results**

#### **Table 12:** Group Interaction P-Values **(FDR Corrected)** for **Blood Biomarkers ~ Cognition * Group**

| Cognitive Score | CD163 (pg/ml) | ICAM (pg/ml) |
| --- | --- | --- |
| EXEZS | 0.38461 | 0.80821 |
| SPEZS | 0.93013 | 0.27136 |
| ATTZS | 0.77051 | 0.98665 |
| LEAZS | 0.71885 | 0.55351 |
| MEMZS | 0.90342 | 0.64763 |
| MOTZS | 0.19725 | 0.95078 |
| LANZS | 0.72652 | 0.54293 |
| TOTALZS | 0.45235 | 0.61067 |

#### **Table 13:** Group Interaction P-Values **(FDR Corrected)** for **Vascular Marker ~ Cognition * Group**

| Cognitive Domain | *r* (HIV+) | *r* (HC) | Interaction *p*-value |
| --- | --- | --- | --- |
| EXEZS | -0.437 | -0.297 | 0.913 |
| SPEZS | -0.220 | -0.322 | 0.427 |
| ATTZS | -0.287 | -0.134 | 0.838 |
| LEAZS | -0.061 | -0.017 | 0.905 |
| MEMZS | -0.097 | 0.057 | 0.610 |
| MOTZS | -0.300 | -0.193 | 0.876 |
| LANZS | -0.653 | -0.007 | *0.062* |
| TOTALZS | -0.462 | -0.166 | 0.439 |

**Supplementary Figures**

**Supplementary Figure 1**

**Supplementary Figure-1:** Combined Brain ROI analysis (WM tracts, GM cortical and Sub-cortical regions) of group interaction for MRE metrics (G- stiffness, phi- viscosity and strain).

**Supplementary Figure 2**

**Supplementary Figure-2:** Interaction of peripheral marker of brain injury (NFL) and Vascular Marker (Reynold’s Risk score) reported with the correlation coefficients of each group and interaction p-value. LEAZS, MEMZS, LANZS– indicates Z-Scores for Learning, Memory and Language Cognitive Tests respectively. HIV+ group indicated in red and HC in blue.
